# Electrophysiological Measures of Tactile and Auditory Processing in Children With Autism Spectrum Disorder

**DOI:** 10.3389/fnhum.2021.729270

**Published:** 2021-12-23

**Authors:** Girija Kadlaskar, Sophia Bergmann, Rebecca McNally Keehn, Amanda Seidl, Brandon Keehn

**Affiliations:** ^1^Department of Speech, Language, and Hearing Sciences, Purdue University, West Lafayette, IN, United States; ^2^Department of Pediatrics, Indiana University School of Medicine, Indianapolis, IN, United States; ^3^Department of Psychological Sciences, Purdue University, West Lafayette, IN, United States

**Keywords:** autism, tactile processing, auditory processing, event-related potentials, oddball paradigm

## Abstract

Behavioral differences in responding to tactile and auditory stimuli are widely reported in individuals with autism spectrum disorder (ASD). However, the neural mechanisms underlying distinct tactile and auditory reactivity patterns in ASD remain unclear with theories implicating differences in both perceptual and attentional processes. The current study sought to investigate (1) the neural indices of early perceptual and later attentional factors underlying tactile and auditory processing in children with and without ASD, and (2) the relationship between neural indices of tactile and auditory processing and ASD symptomatology. Participants included 14, 6–12-year-olds with ASD and 14 age- and non-verbal IQ matched typically developing (TD) children. Children participated in an event-related potential (ERP) oddball paradigm during which they watched a silent video while being presented with tactile and auditory stimuli (i.e., 80% standard speech sound/a/; 10% oddball speech sound/i/; 10% novel vibrotactile stimuli on the fingertip with standard speech sound/a/). Children’s early and later ERP responses to tactile (P1 and N2) and auditory stimuli (P1, P3a, and P3b) were examined. Non-parametric analyses showed that children with ASD displayed differences in early perceptual processing of auditory (i.e., lower amplitudes at central region of interest), but not tactile, stimuli. Analysis of later attentional components did not show differences in response to tactile and auditory stimuli in the ASD and TD groups. Together, these results suggest that differences in auditory responsivity patterns could be related to perceptual factors in children with ASD. However, despite differences in caregiver-reported sensory measures, children with ASD did not differ in their neural reactivity to infrequent touch-speech stimuli compared to TD children. Nevertheless, correlational analyses confirmed that inter-individual differences in neural responsivity to tactile and auditory stimuli were related to social skills in all children. Finally, we discuss how the paradigm and stimulus type used in the current study may have impacted our results. These findings have implications for everyday life, where individual differences in responding to tactile and auditory stimuli may impact social functioning.

## Introduction

Appropriately responding to sensory input is critical for the development of early socio-communicative skills (Bundy et al., 2002). However, atypical sensory processing is reported in 42–95% of individuals with autism spectrum disorder (ASD; Baranek et al., 2006; Leekam et al., 2007; Tomchek and Dunn, 2007; Ben-Sasson et al., 2009; Lane et al., 2011; Marco et al., 2011). These differences in sensory reactivity in ASD are reported in infants (Ben-Sasson et al., 2008), children (Leekam et al., 2007), and adults with ASD (Crane et al., 2009), and are expressed in at least three distinct behavioral response patterns (Dunn, 1997; Baranek et al., 2006; Miller et al., 2007; Ben-Sasson et al., 2009; Ausderau et al., 2014): (1) hyper-reactivity (e.g., exaggerated behavioral responses to sensory stimuli), (2) hypo-reactivity (e.g., reduced or slowed responses to sensory stimuli), and (3) sensory seeking (e.g., engaging in prolonged and intense sensory experiences). Previous studies have shown that different response patterns are present across all sensory modalities in individuals with ASD (Kern et al., 2007; Dudova et al., 2011), however, most studies examining sensory reactivity in ASD have focused on auditory and visual modalities due to their role in socio-communicative development (Bremner et al., 2012) and overall ease of measurement.

Touch, a channel exploited frequently during human interactions (Stack and Muir, 1990), is also used as a social and communicative signal (Hertenstein et al., 2006; Dunbar, 2010). Additionally, touch may impact many aspects of language learning. For example, research has shown that typically developing (TD) infants can use tactile cues presented with speech to find word boundaries (Seidl et al., 2015) and learn abstract patterns (Lew-Williams et al., 2019). Moreover, oral somatosensory feedback has shown to impact speech perception in TD infants (Bruderer et al., 2015). These findings suggest that similar to auditory and visual modalities, the tactile modality may also contribute to the development of socio-communicative skills. To date, few researchers have examined tactile responsivity in children with ASD using observational (Foss-Feig et al., 2012; Baranek et al., 2013; Kadlaskar et al., 2019), psychophysical (O’Riordan and Passetti, 2006; Puts et al., 2014; Tavassoli et al., 2016), or neuroimaging methods (Miyazaki et al., 2007; Marco et al., 2012; Cascio et al., 2015). Collectively, these studies provide mixed results. For example, while observational studies have shown atypical responsivity to touch in individuals with ASD (Baranek et al., 2013; Kadlaskar et al., 2019), studies using psychophysical measures suggest both typical (O’Riordan and Passetti, 2006; Güçlü et al., 2007) and atypical (Puts et al., 2014; Tavassoli et al., 2016) tactile responsivity. For example, Güçlü et al. (2007) examined tactile detection thresholds to 40 and 250 Hz vibrotactile stimuli in 6 children with and without ASD. The results of this study indicated no significant differences between the ASD and control groups in detection thresholds, indicating that children with ASD and TD children show similar perception of tactile information. However, it is possible that the lack of a difference in detection thresholds between groups in Güçlü et al. (2007) may have been a result of low statistical power.

Additional support in favor of equivalent tactile perception comes from O’Riordan and Passetti (2006). They examined 13 high-functioning children with ASD and compared them to 13 TD children on their ability to discriminate the texture of different grades of sandpaper and to detect touches presented with synthetic fibers. Findings of this study indicated no differences in the detection and discrimination abilities between the two groups, suggesting that tactile perception is not different in children with ASD. On the contrary, Puts et al. (2014) showed higher static detection thresholds in children with ASD compared to age and IQ-matched TD controls. Higher static thresholds in children with ASD were also reported in Tavassoli et al. (2016). Additionally, Tavassoli et al. (2016) showed that children with ASD who had higher static detection thresholds also had greater ASD-related traits. Although these studies indicate evidence of atypical tactile responsivity (measured by behavioral responses and tactile detection thresholds) at least in *some* individuals with ASD, they do not highlight underlying mechanisms that may regulate responses to tactile information.

Neuroimaging methods provide a unique opportunity to examine neural processes underlying atypical sensory responsivity. A limited number of neuroimaging studies have examined tactile processing in children with ASD (Miyazaki et al., 2007; Marco et al., 2012; Cascio et al., 2015), and these have also yielded inconsistent results. For example, while Marco et al. (2012) showed that children with ASD show smaller amplitudes of early (S1) responses in the left somatosensory cortex in response to tactile stimuli compared to TD children, Miyazaki et al. (2007) demonstrated enhanced somatosensory processing in even earlier components (N20-P25) in the right hemisphere. In sum, these studies show mixed findings suggesting both hypo- and hyper-responsivity in individuals with ASD. Nonetheless, both studies suggest that sensory behaviors may be related to early perceptual differences in somatosensory processing.

Prior research has also shown links between neural indices of tactile perception and touch responsivity in individuals with ASD. For example, Puts et al. (2017) showed that children with ASD have reduced levels of inhibitory neurotransmitter GABA that are associated with differences in tactile perception. Using MEG, Marco et al. (2012) reported that, participants’ early cortical activity to somatosensory stimuli may be positively related with caregiver-report measures of tactile sensitivity. Additionally, Cascio et al. (2015) found that earlier ERP responses (approximately 120–220 ms post-stimulus) are related to hyper-reactivity in both ASD and TD groups, whereas later ERP components are linked with caregiver-reported measures of hypo-reactivity in the ASD group. The authors conclude that behavioral manifestations of hypo-reactivity in ASD may be related to differences in allocation of attention rather than differences in tactile perception. These findings suggest that atypical allocation of attention may explain at least some of the differences in responding to sensory information in ASD and provide additional insights into the contradictory findings related to hypo- and hyper-responsivity to tactile stimuli. It is possible that, although, some individuals with ASD show no differences in tactile detection thresholds compared to TD children (O’Riordan and Passetti, 2006; Güçlü et al., 2007), atypical behavioral responsivity to touch could be a result of later cortical processes associated with attending to perceived touch.

The mixed evidence in the tactile modality is comparable to the studies examining auditory processing in ASD. For example, using oddball paradigms, some have reported shorter latencies of the N1 ERP component in response to pure tone stimuli in children with ASD (Ferri et al., 2003), which may possibly imply faster processing of basic auditory information. In contrast, others have reported prolonged latencies and smaller amplitudes of the earlier ERP (e.g., P1) and Event Related Field (ERF) components in response to pure and complex tones (Jansson-Verkasalo et al., 2003; Jansson-Verkasalo et al., 2005; Lepistö et al., 2005; Roberts et al., 2010; Donkers et al., 2015) in children with ASD, indicative of slower processing of auditory information in ASD. Evidence of smaller amplitudes and delayed latencies have also been reported in response to speech stimuli (Lepistö et al., 2005; Russo et al., 2009). These results indicate that differences in auditory perceptual processes to both simple and complex stimuli are present in individuals with ASD. Additionally, reduced amplitudes of early ERP/ERF components may reflect higher sensory thresholds for detecting a stimulus, which may result in hypo-reactivity.

While additional studies have provided neuroimaging evidence suggesting atypical perceptual processing of auditory stimuli in ASD (Bruneau et al., 2003; Rosenhall et al., 2003; Fujikawa-Brooks et al., 2010), few researchers have shown equivalent early exogenous responses (measured by P1 amplitudes) to auditory stimuli in ASD and TD groups (Čeponienė et al., 2003). Specifically, using an oddball paradigm, Čeponienė et al. (2003) reported that children with ASD show comparable P1, but atypical P3a amplitudes to auditory stimuli, suggesting differences in involuntary attentional orienting to deviant auditory stimuli. In addition, Whitehouse and Bishop (2008) showed that children with ASD may display both typical and atypical P3a amplitudes to deviant auditory stimuli dependent on the nature of the task (i.e., differences in P3a in passive, but not active tasks) and the type of the stimuli (i.e., differences to speech, but not to non-speech). Together, these findings suggest two possible explanations underlying differences in sensory processing in ASD: (1) the source of sensory processing differences is associated with basic perceptual differences, and/or (2) differences in sensory responsivity may be related to atypical allocation of attention to sensory input. Given these alternate explanations, exploratory hypotheses were formulated.

Our first objective was to examine neural indices associated with perceptual and attentional factors [measured by early (P1) and late ERP (N2, P3a, P3b) components, respectively] underlying tactile and auditory processing in children with ASD compared to their TD peers. We hypothesized that, if differences in behavioral sensory patterns in ASD are related to atypical perception, then differences in early ERP (P1) components in children with ASD would be present. Alternatively, if differences in auditory and tactile processing are related to atypical allocation of attention, then differences between ASD and TD children would be present in later ERP (N2, P3a, P3b) components, associated with attentional processing. Next, because prior research has suggested an association between differences in tactile and auditory responsivity and core features of ASD (Hilton et al., 2010; Foss-Feig et al., 2012; Kargas et al., 2015; Linke et al., 2018; Kadlaskar et al., 2019), our second objective was to examine the relationship between neural indices of tactile and auditory processing and ASD symptomatology (measured using Autism Diagnostic Observation Schedule-2, Social Responsiveness Scale-2, and Sensory Profile-2). We hypothesized that atypical perceptual- and attention-related electrophysiological responses associated with touch-speech processing would be related to increased ASD symptomatology in both ASD and TD groups.

## Materials and Methods

### Participants

Participants included 14 children with ASD and 14 age-, sex-, and non-verbal IQ-matched TD children (6–12 years; Table 1). The age range included in the study was carefully selected to closely match participant characteristics of prior studies that have shown distinct patterns of auditory and tactile ERP responses in children with ASD (Whitehouse and Bishop, 2008; Marco et al., 2012; Cascio et al., 2015). Clinical diagnoses for the ASD group were confirmed using the Autism Diagnostic Observation Schedule, Second Edition (ADOS-2; Lord et al., 2012), the Social Communication Questionnaire (SCQ; Rutter et al., 2003), and expert clinical judgment according to DSM-5. TD participants had no family history of ASD and the absence of clinically significant ASD symptoms was confirmed using parent report (all Total t-scores were below 51 on the Social Responsiveness Scale-2; Constantino, 2012). Handedness was measured using the Edinburgh Handedness Inventory (EHI; Oldfield, 1971). No children in the ASD group reported the presence of other ASD-related medical conditions (e.g., fragile-X syndrome, tuberous sclerosis) or any parent-reported hearing difficulties. Last, 3 additional participants in the ASD group were excluded due to refusal to participate in the electroencephalography (EEG) task (*n* = 2) or excessively noisy EEG data (*n* = 1). All participants and caregivers provided written assent and consent before participating and received monetary compensation as a token of appreciation for their participation. The present research was reviewed and approved by Purdue University Institutional Review Board (IRB).

**TABLE 1:** Participant demographics.

	ASD	TD	Statistic	*p*
N (M:F)	14 (11:3)	14 (11:3)	*X*^2^(1) = 0.00	1.0
Age (years)	10.13 (1.9); 6.17–12.58	9.95 (1.36); 7.78–12.53	*t*(26) = 0.29	0.77
Handedness (R:L)	11:3	13:1	*X*^2^(1) = 1.16	0.28
Verbal IQ	98 (21); 67–126	117 (11); 94–135	*t*(26) = −2.97	0.006
Non-verbal IQ	108 (18); 74–136	117 (16); 89–144	*t*(26) = −1.46	0.15
**ADOS-2**				
Social affect	10 (5); 4–20	−	−	−
Repetitive behavior	3 (2); 1–6	−	−	−
Severity scores	8 (2); 4–10	−	−	−
**SRS-2**				
SCI	78 (8.03); 62–90	43.28 (4.85); 36–49	*t*(26) = 13.82	< 0.001
RRB	78.42 (9.33); 66–90	45 (4.4); 41–55	*t*(26) = 12.11	< 0.001
Total	78.92 (7.75); 66–90	43.64 (4.43); 37–51	*t*(26) = 14.78	< 0.001
**SP-2**				
Touch raw score	24 (9); 5–41	10 (5); 0–15	*t*(26) = 4.96	< 0.001
Auditory raw score	28 (7); 15–38	12 (4); 2–21	*t*(26) = 7.41	< 0.001
**Usable trials (N)**				
Standard	552 (150); 368–857	654 (161); 405–871	*t*(26) = −1.73	0.09
Oddball	71 (21); 46–107	83 (21); 49–112	*t*(26) = −1.45	0.15
Novel	69 (21); 36–108	84(20); 49–109	*t*(26) = −1.99	0.06

*ADOS-2, Autism Diagnostic Observation Schedule-2; SRS-2; Social Responsiveness Scale-2; SP-2, Sensory Profile 2.*

*IQ-based matching was determined using the Wechsler Abbreviated Scale of Intelligence (ASD 10, TD 8; WASI-II; Wechsler, 2011) or the Differential Ability Scale II (ASD 4, TD 6; DAS-II; Elliott, 2007); Verbal and Non-verbal IQ scales of the WASI-II and DAS-II are highly correlated (Elliott, 2007). Mean (SD); range.*

### Standardized Measures

#### Autism Diagnostic Observation Schedule-2

The ADOS-2 (Lord et al., 2012) is a semi-structured, standardized assessment of communication, social interaction, play, and restricted and repetitive behaviors. In the present study, all children in the ASD group were administered ADOS-2 Module 3 which is appropriate for children and adolescents with fluent speech. ADOS-2 calibrated severity scores (CSS) were used as a measure of ASD symptom severity, with higher CSS scores reflecting greater severity (Gotham et al., 2009).

#### Social Responsiveness Scale-2

The SRS-2 is a caregiver-report questionnaire that provides a quantitative measure of autism-related traits during the past 6 months. The School-Age form was completed by caregivers in both the ASD and TD groups. The SRS-2 Total scores as well as Social Communication and Interaction (SCI) and Restricted Interests and Repetitive Behavior (RRB) scores were used as measures of ASD symptom severity, with higher scores reflecting greater severity.

#### Sensory Profile-2

The SP-2 (Dunn, 2014) is a caregiver-report questionnaire that assesses everyday sensory processing in 3–14-year-olds. It is divided into six sensory domains (auditory, visual, touch, movement, body position, and oral), three behavioral categories (conduct, social emotion, and attention), and four quadrants (seeking, avoiding, sensitivity, and registration). Touch and Auditory Sensory Profile scores were used as measures of tactile and auditory sensory processing, respectively.

### Experimental Stimuli

#### Auditory Stimuli

Auditory stimuli consisted of two vowels (/a/and/i/) generated using Praat software (Boersma and Weenink, 2019). Stimuli displayed a fundamental frequency of 140 Hz (as it fits within the pitch range of a typical male speaker; Goy et al., 2013), and lasted for 200 ms (similar to the duration of stimuli used in Whitehouse and Bishop, 2008). Auditory stimuli were presented at 60 dB using a display-integrated speaker (ViewSonic VG732m).

#### Tactile Stimuli

**FIGURE 1:**
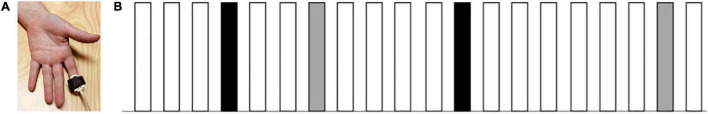
**(A)** Mechanical tactor used to deliver the vibrotactile stimuli. **(B)** Illustration of the oddball paradigm. White bars represent the standard stimuli/a/ (80%), black bars represent the oddball stimuli/i/ (10%), and gray bars represent the novel vibrotactile stimuli on the fingertip of the index finger along with the speech sound/a/ (10%). Stimuli were presented randomly with at least two standard stimuli prior to every oddball or novel stimuli. Each tactile and auditory stimulus lasted for 200 ms (ISI = 1,400 ms).

A customized tactor was used to deliver vibrotactile stimuli to the index fingertip of each participant’s non-dominant hand (Figure 1). The location of the vibrotactile stimuli was consistent with past studies that have examined touch responsivity in individuals with ASD (Blakemore et al., 2006; Marco et al., 2012; Cascio et al., 2015). Vibrotactile stimulation was presented at 290 Hz, as individuals with ASD have shown differences in tactile responsivity to high-frequency, but not low-frequency, vibrations (Blakemore et al., 2006). Each participant’s hand was covered with a towel to mask the sound coming from the tactor, and also because seeing somatosensory stimulation has been shown to modulate somatosensory cortical responses (Taylor-Clarke et al., 2002). All tactile stimuli lasted for 200 ms.

### Procedure

To ensure the cooperation of all participants, the total testing time was divided into two sessions. The first session included consenting and standardized testing, and the second session included EEG data collection. EEG data were collected in a dimly lit room. Participants were seated at a conformable viewing distance of approximately 60 cm from a computer monitor and speaker. Prior to applying the EEG net, the child’s head was measured, and a small mark was made at the top of the participant’s head to allow proper placement of the net. Before the net was placed over the participant’s head, sponges to be used were soaked in a salt-water solution [distilled water + potassium chloride (KCl) + baby shampoo]. After the net application, the tactor was placed on the participants’ index fingertip of the non-dominant hand and was covered with a hand towel to mask the sound coming from the tactor and viewing of the tactor. Participants were instructed to sit still throughout the duration of the experiment. A trained research assistant sat behind the participant to ensure that participants were following the instructions. Next, a passive auditory oddball paradigm was employed. Participants watched a silent video of their choice (e.g., Fining Nemo, The Good Dinosaur, Shrek, Cars 2 etc.) on the computer monitor and were presented with auditory stimuli consisting of 80% of the standard stimuli (the speech sound/a/), 10% of the oddball stimuli (the speech sound/i/), and 10% of the novel stimuli (vibrotactile stimulation on the fingertip of the index finger along with the standard speech sound/a/; Figure 1). The task contained 1,200 trials in total, which were divided into 4 blocks of 300 trials each. The stimuli were presented randomly (ISI = 1,400 ms) with at least two standard stimuli prior to every oddball and novel stimuli. In all four blocks, participants were instructed to watch the movie and ignore the sounds and the “tingles.”

### Electroencephalography (EEG)

#### EEG Acquisition

EEG data were recorded using 124 or 128-channel HydroCel Geodesic Sensor Nets (HCGSN, Electrical Geodesics, Inc., Eugene, OR) with NetAmps 400 amplifier. Electrooculography (EOG) electrodes (i.e., 125, 126, 127, 128) in a 128-channel net were excluded from data collection because EOG electrodes that are usually placed on the participant’s face may reduce compliance in participants. EEG data were recorded in Net Station 5.2 software (HCGSN, Electrical Geodesics, Inc., Eugene, OR). The continuous EEG data were digitized at 500 Hz and referenced online to the vertex (electrode Cz). Impedances were kept below 100 kΩ. A 0.1 Hz high-pass filter was applied to the raw data, which was subsequently segmented into 1,100 ms epochs (100 ms pre- and 1,000 ms post-stimulus onset).

#### EEG Pre-processing

EEG data processing was completed offline using a MATLAB-based toolbox EEGLAB (Delorme et al., 2011). First, the raw EEG data were digitally filtered using a 0.5–50 Hz bandpass filter. Epochs in each channel were marked bad if they had amplitude values exceeding ± 150 μV. Subsequently, channels were marked bad if they had more than 25% of epochs rejected. Manual artifact detection was then carried out on continuous EEG data to reject non-stereotyped artifacts. After filtering and removal of non-stereotyped artifacts, Independent Component Analysis (ICA; Jung et al., 2000) was carried out in EEGLAB. SASICA (Semi-Automated Selection of Independent Components of the electroencephalogram for Artifact correction) was then used to identify artifacts associated with eye movements, saccades, muscle contractions, and bad channels (Chaumon et al., 2015). After removing artifactual independent components, bad channels were replaced using spherical interpolation, and data were re-referenced to the average reference. Artifact-corrected data were segmented into epochs 100 ms prior to and 1,000 ms after the onset of the stimulus for each stimulus type (standard, oddball, novel), baseline corrected, epochs containing residual artifacts were rejected (±150 μV), and any remaining channels with more than 25% of bad epochs were interpolated.

Before analyses, participants (*n* = 1; ASD) with fewer than 20 usable trials in each of the stimulus types (standard, oddball, novel) were excluded. The decision to exclude participants with fewer than 20 usable trials was based on past research requiring a minimum of 10 usable trials in each condition to be included in the final sample (Wagner et al., 2013).

#### Event-Related Potential (ERP) Processing

ERP data processing was completed using ERPLAB toolbox (Lopez-Calderon and Luck, 2014) in MATLAB. Following filtering, artifact correction and rejection, and re-referencing to average reference, averaged ERPs from accepted epochs were created for each stimulus type (standard, oddball, novel). Next, regions of interest (ROIs) were generated using 5 clusters of EGI HydroCel GSN electrodes in the frontal (11, 4, 19, 16), central (129, 55, 106, 7), and parietal (62, 67, 72, 77), and left-central (35, 40, 41 36) and right-central (104, 103, 109, 110) regions corresponding to Fz, Cz, Pz, C3, and C4 locations, respectively. In order to examine tactile perceptual components, we primarily focused on left-central and right-central ROIs. These ROIs were chosen to examine ispi- and contralateral activation in response to tactile stimulation. Auditory perceptual components were examined by analyzing frontal, central and parietal ROIs. These ROIs were chosen based on previous evidence showing that early auditory responses are observed over the midline in the frontocentral regions (Picton et al., 1974; Whitehouse and Bishop, 2008; Donkers et al., 2015). Next, in order to examine attentional components, we primarily focused on the frontal ROI for the novel stimuli and both frontal and parietal ROI for the oddball stimuli. These ROIs were chosen based on past evidence showing that attentional capture to novel stimuli is observed in the frontocentral regions, whereas, attentional capture to oddball stimuli is observed over the frontal and parietal regions (Katayama and Polich, 1998; Polich, 2003).

Selection of specific time windows for analyzing ERP components was based on past research showing that early ERP components reflect basic sensory processing (Ponton et al., 2000) as well as conscious perception (P100; Schubert et al., 2006), whereas later ERP components are more likely to be affected by attention (Novelty N2; Schomaker and Meeter, 2014) and may reflect cognitive processing underlying deviant (P3a, P3b) stimuli (Polich, 2003). Early and late ERP components were identified by visually inspecting the scalp maps as well as the grand-averaged waveforms to novel and oddball stimuli. Mean amplitude was calculated for P1 (80–180 ms), N2 (250–400 ms), P3a (250–500 ms), and P3b (500–700 ms). Finally, as part of our exploratory analyses we also calculated P1 (80–180 ms) at frontal, central, and parietal ROIs for standard stimuli to examine fundamental differences in auditory processing between the ASD and TD groups.

Last, we calculated difference waves to examine the changes in amplitudes as a result of receiving novel and oddball stimuli in a stream of standard stimuli. Difference waves were calculated by subtracting ERP standard waveforms from novel and oddball ERP waveforms. Mean amplitudes of the difference waves were then calculated for N2 between 250 and 400 ms post stimulus, P3a between 250 and 500 ms post stimulus, and for P3b between 500 and 700 ms post stimulus.

## Results

Independent-samples *t*-tests showed a greater number of parent-reported sensory symptoms in tactile and auditory domains in children with ASD compared to TD children (Table 1). The two groups did not differ significantly in the number of usable trials for standard, oddball, and novel stimuli across the four blocks (Table 1), and there was no significant correlation between the number of usable trials and the mean amplitude of ERP components of interest (all *ps* > 0.19).

Given our relatively small sample size, non-parametric tests conducted in SPSS Statistics (version 27) were used. Despite the advantages of non-parametric tests with small sample sizes, these tests are considered less powerful than parametric tests. Therefore, we elected to supplement our analysis using Bayesian statistics (JASP, 2020) as Bayes Factor values indicate the strength of evidence in favor of both the null and alternate hypotheses.

### Novel Tactile Stimuli

#### Early ERP Responses (P1)

**FIGURE 2:**
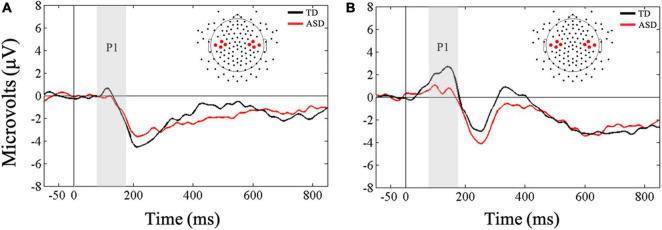
Grand averaged ERPs from **(A)** ipsilateral and **(B)** contralateral stimulation sites for novel tactile stimuli. Gray bars reflect our window of analysis for P1 (80–180 ms post stimulus onset).

Friedman’s test was conducted to examine whether there was a within-subjects main effect of ROI (ipsilateral, contralateral) in response to vibrotactile stimulation. As expected, results showed that there was a significant difference in mean amplitudes between ipsilateral and contralateral ROIs, *X*^2^(1) = 7, *p* = 0.008, with greater amplitude in the contralateral compared to the ipsilateral ROI in response to tactile stimulation for all children (Figure 2). These results were supported by Bayesian analysis (BF_10_ = 67.13) indicating strong evidence in support of the finding. Next, a Mann-Whitney *U*-test was conducted to examine between-group differences in contralateral and ipsilateral ROIs. Results showed that the two groups did not differ in their mean amplitudes in response to novel tactile stimulation at both the contralateral (*U* = 74, *p* = 0.27, *r* = 0.20) and ipsilateral (*U* = 87, *p* = 0.61, *r* = 0.09) ROIs. However, Bayesian *t*-tests showed only anecdotal evidence in support of the null hypotheses for both the contralateral and ipsilateral ROIs (BF_10_ = 0.96; BF_10_ = 0.41, respectively).

#### Late ERP Responses (N2)

A Friedman’s test was conducted to examine whether there was a within-subjects main effect of ROI (frontal, central, parietal) in response to vibrotactile stimulation. Results confirmed the main effect of ROI, *X*^2^(2) = 33.07, *p* < 0.001, and as expected, follow-up Wilcoxon signed-rank tests showed more negative mean amplitudes in the frontal ROI compared to central (*Z* = −3.93, *p* < 0.001, *r* = 0.52) and parietal (*Z* = −4.52, *p* < 0.001, *r* = 0.60) ROIs in response to tactile stimulation. Supplementary Bayesian paired-samples *t*-tests confirmed that N2 amplitudes in response to novel tactile stimulation were greater in the frontal ROI compared to the central (BF_10_ > 100) and parietal ROIs (BF_10_ > 100).

**FIGURE 3:**
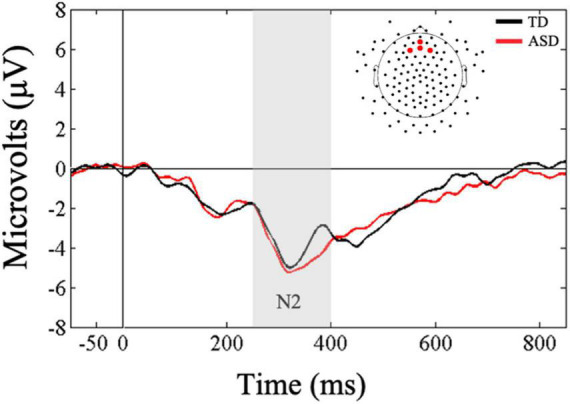
Grand averaged ERPs from frontal regions of interest for novel tactile stimuli. Gray bar reflects our window of analysis for N2 (250–400 ms post stimulus onset).

Next, a Mann-Whitney *U*-test was conducted to examine between group differences in mean N2 amplitudes to novel tactile stimuli in the frontal ROI. Results showed that the two groups did not differ in their amplitudes in response to touch (*U* = 69, *p* = 0.18, *r* = 0.25; Figure 3). These results were further supported by our difference wave analysis using a Mann-Whitney *U*-test (*U* = 82, *p* = 0.46, *r* = 0.13), showing similar mean N2 amplitudes in the ASD and TD groups. However, Bayesian *t*-tests examining between-group differences in mean N2 amplitudes as well as difference wave analysis showed only anecdotal evidence in support of the null hypothesis (BF_10_ = 0.61; BF_10_ = 0.45, respectively).

### Auditory Oddball Stimuli

#### Early ERP Responses (P1)

**FIGURE 4:**
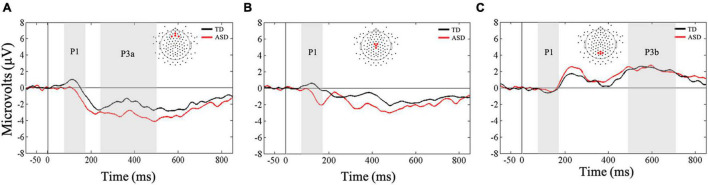
Grand averaged ERPs from **(A)** frontal, **(B)** central, and **(C)** parietal regions of interest for auditory oddball stimuli. Gray bars reflect our window of analysis for P1 (80–180 ms post stimulus onset), P3a (250–500 ms post stimulus onset), and P3b (500–700 ms post stimulus onset).

Friedman’s test was conducted to examine whether there was a within-subjects main effect of ROI (frontal, central, parietal) in response to the auditory oddball stimuli. Results indicated that there was no main effect of ROI, *X*^2^(2) = 0.50, *p* < 0.77. Bayesian analysis confirmed that mean auditory P1 amplitude did not differ as a result of ROI (BF_10_ = 0.12; substantial evidence for the null hypothesis). Next, Mann-Whitney *U*-tests were conducted to examine whether the two groups differed significantly in their early responsivity to oddball stimuli at all three ROIs. Results showed that children in the ASD group showed significantly lower mean amplitudes at central (*U* = 51, *p* = 0.03, *r* = 0.40), but not the frontal (*U* = 58, *p* = 0.07, *r* = 0.34) or parietal (*U* = 76, *p* = 0.31, *r* = 0.19) ROIs compared to the TD group, suggesting that the two groups may differ in their early perceptual responses to auditory oddball stimuli and this difference may be observed over the central ROI (Figure 4). Bayesian *t*-tests provided substantial evidence that children with ASD showed reduced mean P1 amplitudes at central ROI compared to TD children (BF_10_ = 3.09). Additionally, there was anecdotal evidence suggesting lower mean P1 amplitudes in the ASD group at frontal ROI (BF_10_ = 1.44) and similar mean P1 amplitudes at parietal ROIs (BF_10_ = 0.52) compared to the TD group.

#### Late ERP Responses (P3a, P3b)

First, a Mann-Whitney *U*-test was conducted to examine between-group differences in mean P3a amplitudes in the frontal ROI. Results showed that the two groups did not differ in their mean amplitudes in responses to the auditory oddball stimuli (*U* = 62, *p* = 0.10, *r* = 0.31; Figure 4). Our Bayesian analysis supported these findings (BF_10_ = 0.93). However, the difference wave analysis showed that the TD group exhibited greater changes in amplitude as a result of receiving oddball stimuli in a stream of standard stimuli compared to the ASD group (*U* = 53, *p* = 0.03, *r* = 0.39). Nevertheless, these results were not supported by our Bayesian analysis that only showed anecdotal evidence in support of the finding (BF_10_ = 1.26).

Next, a Friedman’s test was conducted to examine whether there was a within-subjects main effect of ROI (frontal, central, parietal) in response to the auditory oddball stimuli. Results confirmed the main effect of ROI, *X*^2^(2) = 40.5, *p* < 0.001, and as expected, follow-up Wilcoxon signed-rank tests showed greater mean amplitudes in the parietal ROI compared to frontal (*Z* = −4.55, *p* < 0.001, *r* = 0.60) and central (*Z* = −4.62, *p* < 0.001, *r* = 0.61) ROIs in response to the oddball stimuli. Bayesian paired-samples *t*-tests confirmed that mean P3b amplitudes in response to the auditory oddball stimuli were greater in parietal ROI compared to the frontal (BF_10_ > 100) and central ROIs (BF_10_ > 100). These results were further supported by our difference wave analysis using a Mann-Whitney *U*-test.

Next, a Mann-Whitney *U*-test was conducted to examine between group differences in mean P3b amplitudes in the parietal ROI. Results showed that the two groups did not differ in their mean amplitudes in responses to the auditory oddball stimuli (*U* = 85, *p* = 0.55, *r* = 0.11; Figure 4). These results were further supported by our difference wave analysis using a Mann-Whitney *U*-test (*U* = 95.5, *p* = 0.90, *r* = 0.02). However, Bayesian *t*-tests examining mean P3b amplitudes as well as difference wave analysis showed only anecdotal evidence in support of the null hypothesis (BF_10_ = 0.36; BF_10_ = 0.35, respectively).

### Exploratory Analysis: Standard Stimuli

#### Early ERP Responses (P1)

**FIGURE 5:**
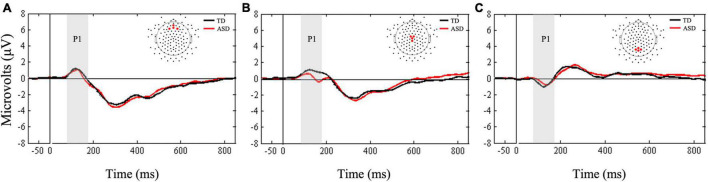
Grand averaged ERPs from **(A)** frontal, **(B)** central, and **(C)** parietal regions of interest for standard stimuli. Gray bars reflect our window of analysis for P1 (80–180 ms post stimulus onset).

A Friedman’s test was conducted to examine whether there was a within-subjects main effect of ROI (frontal, central, parietal) in response to the standard stimuli. Results confirmed the main effect of ROI, *X*^2^(2) = 10.5, *p* = 0.005. Follow-up Wilcoxon signed-rank tests showed more positive mean amplitudes in the frontal and central ROIs compared to the parietal ROI (*Z* = −2.98, *p* < 0.003, *r* = 0.19; *Z* = −3.27, *p* < 0.001, *r* = 0.61, respectively, Figure 5). Supplementary Bayesian paired-samples *t*-tests confirmed that mean P1 amplitudes underlying standard stimuli were greater in the frontal (BF_10_ = 20.05) and central (BF_10_ > 100) ROIs compared to the parietal ROI.

Next, Mann-Whitney *U*-tests were conducted to examine between group differences in mean P1 amplitudes to standard stimuli. The two groups did not differ in mean amplitudes at any of the three ROIs (all *ps* > 0.14). Bayesian *t*-tests supported these results by showing only anecdotal evidence in support of the null hypotheses for frontal and parietal ROIs (BF_10_ = 0.39; BF_10_ = 0.37, respectively). Finally, there was anecdotal evidence suggesting that mean P1 amplitudes at central ROI were greater in the TD group compared to the ASD group (BF_10_ = 1.14).

### Association Between Neural Indices and ASD Symptomatology

Spearman’s correlations were conducted to examine the association between mean amplitudes of tactile (P1, N2) and auditory (P1, P3b) ERP components with ADOS-2, SRS-2, and tactile and auditory subscales of SP-2.

#### Neural Responsivity to Touch and ASD Symptomatology

**FIGURE 6:**
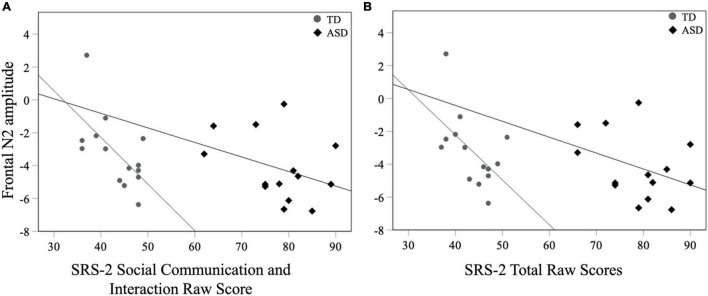
Mean frontal N2 amplitudes to novel stimuli and SRS-2 **(A)** SCI and **(B)** Total scores for both groups.

No correlations between contralateral mean P1 in response to touch and ADOS-2, SRS-2, and the tactile subscale of the SP-2 were significant (all *ps* > 0.08). For later neural indices, there was a significant negative correlation between mean frontal N2 amplitudes and the SCI score of the SRS-2 [*r*_*s*_ (28) = −0.41, *p* = 0.02] as well as the Total score [*r*_*s*_ (28) = −0.42, *p* = 0.02] for all participants with larger negative N2 amplitudes associated with greater SRS-2 scores (Figure 6). There were no correlations between mean N2 amplitudes and other measures such as the ADOS-2 and the tactile subscale of the SP-2 (all *ps* > 0.06; Table 2).

**TABLE 2:** Correlations between mean N2 amplitude and ASD symptomatology.

	ADOS-2			SRS-2			*SP*−2
Group	SA	RRB	SS	SCI	RRB	Total	Touch
All (*n* = 28)	−	−	−	−0.41*	–0.24	−0.42*	–0.16
ASD (*n* = 14)	0.32	0.26	0.27	–0.31	–0.07	–0.33	–0.18
TD (*n* = 14)	−	−	−	–0.50	0.06	–0.49	0.18

*SA, Social Affect; RRB, Restricted and Repetitive Behaviors; SS, Severity Score; SCI, Social Communication and Interaction; RRB, Restricted Interests and Repetitive Behaviors; Touch, Touch section of Sensory Profile-2.*

*p < 0.05.

#### Neural Responsivity to Oddball Stimuli and ASD Symptomatology

**FIGURE 7:**
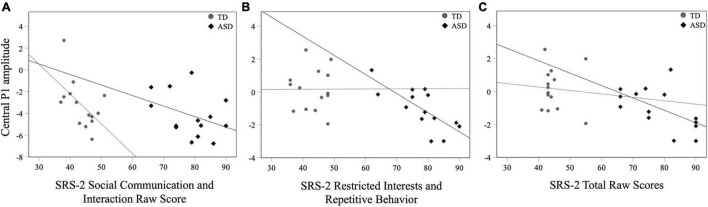
Mean central P1 amplitudes to oddball stimuli and SRS-2 **(A)** SCI, **(B)** RRB, and **(C)** Total scores for both groups.

**FIGURE 8:**
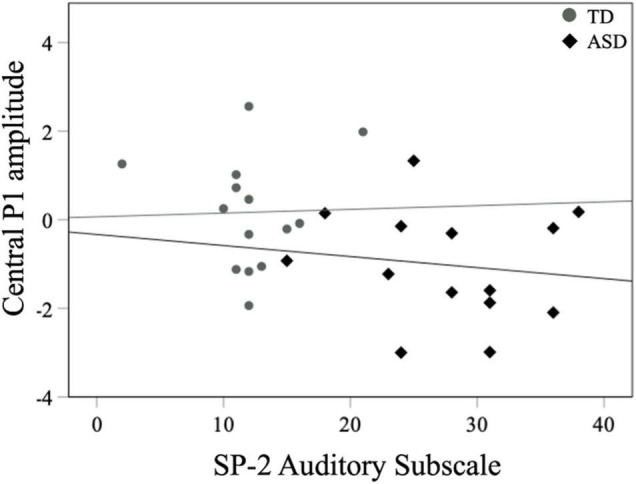
Mean central P1 amplitudes to oddball stimuli and SP-2 auditory subscale scores for both groups.

**FIGURE 9:**
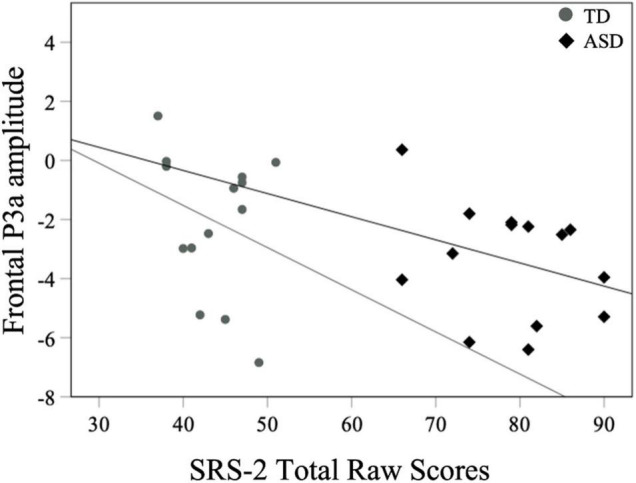
Mean frontal P3a amplitudes to oddball stimuli and SRS-2 scores for both groups.

For all children, mean central P1 amplitudes to oddball stimuli were negatively correlated with the SRS-2 Total score [*r*_*s*_ (28) = −0.56, *p* = 0.002], suggesting that reduced P1 amplitudes were associated with increased social challenges. Additionally, for all children, mean P1 amplitudes to oddball stimuli were also negatively correlated with both the subscales of the SRS-2 [SCI; *r*_*s*_ (28) = −0.54, *p* = 0.003, RRB; *r*_*s*_ (28) = −0.52, *p* = 0.004; Figure 7]. A similar relationship between mean P1 amplitudes to oddball stimuli and SRS-2 scores was observed for children in the ASD, but not the TD, group. Next, for all children, a significant negative correlation was obtained between mean P1 amplitudes and the SP-2 Auditory subscale [*r*_*s*_ (28) = −0.40, *p* = 0.03; Figure 8], suggesting that reduced mean P1 amplitudes may be associated with increased parent-reported auditory differences. Mean P1 amplitudes to oddball stimuli were not correlated with the ADOS-2 scores (all *p*s > 0.37; Table 3). Next, for all children, mean P3a amplitude was negatively correlated with SRS-2 Total score [*r*_*s*_ (28) = −0.39, *p* = 0.04; Figure 9 and Table 4], suggesting that reduced P3a amplitudes were associated with increased social challenges. Finally, there was no correlation between mean P3b amplitude and any of our measures (all *p*s > 0.05).

**TABLE 3:** Correlations between auditory mean central P1 amplitude and ASD symptomatology.

	ADOS-2			SRS-2			*SP*−2
Group	SA	RRB	SS	SCI	RRB	Total	Auditory
All (*n* = 28)	−	−	−	−0.54**	−0.52**	−0.56**	−0.40*
ASD (*n* = 14)	−0.25	0.01	−0.24	−0.74**	−0.65*	−0.82**	–0.12
TD (*n* = 14)	−	−	−	0.04	–0.05	–0.007	–0.12

*SA, Social Affect; RRB, Restricted and Repetitive Behaviors; SS, Severity Score; SCI, Social Communication and Interaction; RRB, Restricted Interests and Repetitive Behaviors; Auditory, Auditory section of Sensory Profile-2.*

*p < 0.05; **p < 0.01.

**TABLE 4:** Correlations between auditory mean frontal P3a amplitude and ASD symptomatology.

	ADOS-2			SRS-2			*SP*−2
Group	SA	RRB	SS	SCI	RRB	Total	Auditory
All (*n* = 28)	−	−	−	–0.35	–0.34	−0.39*	–0.18
ASD (*n* = 14)	0.02	0.48	−0.01	–0.19	–0.17	–0.29	–0.05
TD (*n* = 14)	−	−	−	–0.20	–0.08	–0.25	0.26

*SA, Social Affect; RRB, Restricted and Repetitive Behaviors; SS, Severity Score; SCI, Social Communication and Interaction; RRB, Restricted Interests and Repetitive Behaviors; Auditory, Auditory section of Sensory Profile-2.*

*p < 0.05.

#### Neural Responsivity to Standard Stimuli and ASD Symptomatology

There were no correlations between mean standard P1 amplitude and ADOS-2 scores, SRS-2 scores, and SP-2 auditory subscale score (all *p*s > 0.09).

## Discussion

The present study assessed neural indices of perceptual and attentional factors underlying tactile and auditory processing in children with ASD. Specifically, we examined (1) whether atypical responsivity to tactile and auditory stimuli in children with ASD is related to differences in perceptual or attentional processing compared to TD children, and (2) the relationship between neural indices of tactile and auditory processing and ASD symptomatology in children with and without ASD.

### Neural Indices Underlying Tactile Processing

As expected for the tactile modality, all children showed increased P1 amplitude at the contralateral compared to the ipsilateral location of the stimulation. This finding supports previous studies that have shown greater early contralateral activation in response to touch in ASD and TD (Russo et al., 2010; Cascio et al., 2015). However, P1 amplitudes at contralateral and ipsilateral locations did not differ across groups. Likewise, for later neural responses, children with ASD and TD showed similar N2 amplitudes in response to touch. This finding stands in contrast to previous ERP/ERF studies (Russo et al., 2010; Marco et al., 2012) suggesting diminished early cortical responses to touch in individuals with ASD. One possible reason for these mixed findings could be attributed to methodological differences between studies. For example, in Marco et al. (2012), both standard and oddball tactile stimuli consisted of pneumatically-driven pulses, whereas in the current study tactile stimuli consisted of vibrations presented only as novel stimuli embedded in a stream of auditory sounds. It is possible that the change in modality in our novel stimuli may have facilitated neural responsivity in individuals with ASD.

Additionally, tactile stimulation in our study was always presented together with an auditory stimulus. Although there are differences in auditory-somatosensory integration in children with ASD (Russo et al., 2010), it is possible that the presentation of bimodal input streams may have facilitated neural responsivity to novel stimuli. Support for this argument comes from previous research that has shown diminished amplitudes in response to unimodal somatosensory input starting around 70 ms post-stimulus onset in children with ASD compared to TD children; however, the authors do not present a detailed discussion on amplitude differences between unimodal and bimodal stimuli (Russo et al., 2010). Benefits of intersensory processing are also observed in TD infants using other modalities. For example, Reynolds et al. (2014) showed that bimodal audio-visual stimuli with intersensory redundancies are linked with increased neural responsiveness (higher amplitude in midline negative wave) in 5-month-olds. The authors further argue that this increased neural responsivity associated with intersensory redundancy may indicate greater attentional salience and more efficient processing of incoming stimuli in young infants. The impact of bimodal input on sensory responsivity as measured by orienting responses has been observed in a behavioral study (Kadlaskar et al., 2020), where infants at risk for ASD showed more attentional orienting to bimodal touch-speech input compared to unimodal touch-only input presented by their mothers. However, further research is needed to investigate whether the presentation of tactile input along with auditory input facilitated neural responsivity in the ASD group.

Although the current study did not find neurophysiological differences in processing tactile stimuli between ASD and TD groups, these differences were still present at a behavioral level as measured by the SP-2 (Table 1). A possible reason for the discrepancy between our neural and parent-report measures could be attributed to our relatively small sample size. Additionally, selection of stimulus type (non-human vibrotactile stimuli) and/or paradigm (passive task) may have contributed to these inconsistent findings. Future studies should further investigate this topic using large sample sizes and exploring how a variety of tactile stimuli (e.g., pressure, air puff, human touch etc…) and paradigms (active vs. passive tasks) may impact our understanding of the relationship between behavioral and neurological markers of sensory processing and attention.

### Neural Indices Underlying Auditory Processing

Our results related to auditory stimuli showed that children with ASD displayed reduced amplitudes in early ERP responses over the central region in response to oddball stimuli compared to the control group, suggesting that differences in responding to infrequent auditory stimuli may be related to atypical perceptual processes in children with ASD. These findings support previous studies that have shown atypical early cortical responsivity to auditory stimuli in ASD (Bruneau et al., 2003; Rosenhall et al., 2003; Fujikawa-Brooks et al., 2010).

Next, while our difference wave analysis for P3a showed that children with ASD exhibited reduced changes in amplitudes as a result of receiving infrequent oddball stimuli embedded in a stream of standard stimuli, there results were not supported by our supplementary Bayesian analysis. Therefore, although this finding suggests some differences in attentional processing of auditory oddball stimuli, these results need to be replicated in future studies using similar paradigms. For P3b, the ASD and TD groups did not differ in their ERP amplitudes to auditory oddball stimuli. Together P3a and P3b findings partially contradict previous studies that have found attenuated later neural responses to auditory stimuli in ASD (Čeponienė et al., 2003; Lepistö et al., 2005; Whitehouse and Bishop, 2008; Donkers et al., 2015). Our results also did not find between group differences in early ERP responses to standard stimuli. Discrepancies among results could be attributed to differences in participant characteristics [e.g., in Lepistö et al. (2005) mean VIQ for the ASD group was 59, whereas in the current study it was 98] and/or the nature of the stimuli (e.g., speech vs. tones). These results indicate that behavioral differences in responding to infrequent auditory stimuli *may* be related to atypical perceptual processing and may contribute to the observed phenotype. However, given the inconsistent findings underlying the P3a, more research is needed to understand how later attentional factors may impact auditory processing in ASD and TD groups.

Note that, the null results in early [observed in the tactile (P1) modality] and late ERP components [observed in both the tactile (N2) and auditory (P3b) modalities] between ASD and TD groups only received anecdotal support from Bayesian analyses. Consideration of Bayesian results is important because in frequentist statistics, results with non-significant *p*-values do not by default confirm the null hypothesis, but instead only show inconclusive evidence to reject the null hypothesis (Kim and Bang, 2016). Because Bayesian analysis only showed anecdotal evidence that the two groups did not differ in their neural responsivity to certain sensory stimuli, it becomes imperative to extend these findings with larger sample sizes to examine generalizability of our findings.

Next, correlational analyses partially supported our hypothesis that neural correlates of tactile and auditory processing would be related to ASD symptomatology in ASD and TD groups. Contrary to our hypothesis, early contralateral responses to touch were not associated with tactile SP-2 scores for all children. However, in the auditory modality, early amplitudes underlying oddball stimuli were associated with parent-reported auditory reactivity and social skills for all children with reduced neural responses being related to increased auditory symptoms as well as with differences in reciprocal social communication. A significant relationship between early auditory ERP responses and social communication was also present for the ASD group. These findings support previous research showing associations between sensory symptoms and ASD symptomatology (Watson et al., 2011; Foss-Feig et al., 2012). Later ERP responses to touch and speech were associated with social measures for all children. Specifically, greater amplitudes at N2 were associated with higher SRS-2 Total and SCI scores. These findings extend previous research suggesting possible links between neural hyper-reactivity to novel stimuli and ASD-related traits (Gomot et al., 2008). Additionally, P3a amplitudes underlying oddball stimuli were associated with SRS-2 Total scores for all children suggesting that links between patterns of allocation of attention to infrequent stimuli and ASD-like traits, regardless of clinical diagnosis, may represent a more dimensional characterization of ASD. Finally, late neural responses to auditory stimuli measured by P3b were not associated with ASD symptomatology.

This study is limited by a few key factors. First, our sample was relatively small given limited data collection due to COVID-19. Second, our ASD sample only included high-functioning children with ASD, and as a result, may not be adequately representative of a more heterogenous sample of ASD. Third, our paradigm did not include presentation of tactile-only input to examine whether neural responsivity to touch observed in our study was impacted by the presence of accompanying auditory input. Finally, the rationale for the present study was based on the premise that earlier ERP components indicate perceptual processing of incoming stimuli, whereas later ERP components suggest higher-order cognitive functioning. However, we acknowledge that in certain contexts attention may also modulate early sensory processing in typical development (Eimer and Forster, 2003). Future studies should use larger sample sizes and alternative task designs to examine how attention may impact early neural responsivity to tactile and auditory stimuli in ASD. Future studies could also consider examining neural responsivity to touch using both unimodal and bimodal input.

In sum, the present study revealed that despite differences in caregiver-reported sensory measures, children with ASD did not differ in their neural reactivity to infrequent touch-speech bimodal stimuli compared to TD children. Our study also showed that, differences in behavioral patterns of auditory responsivity in ASD could be related to perceptual factors in children with ASD. Finally, individual differences in neural responsivity to tactile and auditory stimuli may be related to social skills in all children. Overall, our study suggests that perceptual and attentional factors may differently impact behavioral responses to sensory stimuli. Therefore, examining early and late neural processing to sensory stimuli presented in variety of contexts could be beneficial in future research that aims to study sensory processing using dimensional characterization of ASD.

## Data Availability Statement

The raw data supporting the conclusions of this article will be made available by the authors, without undue reservation.

## Ethics Statement

The studies involving human participants were reviewed and approved by the Institutional Review Board of Purdue University. Written informed consent to participate in this study was provided by the participants’ legal guardian/next of kin.

## Author Contributions

GK, BK, and AS conceived of the study, analyzed the data, and drafted the initial version of the manuscript. GK, SB, RM, and BK collected the data. All authors reviewed and revised the manuscript and approved the final version of the manuscript.

## Conflict of Interest

The authors declare that the research was conducted in the absence of any commercial or financial relationships that could be construed as a potential conflict of interest.

## Publisher’s Note

All claims expressed in this article are solely those of the authors and do not necessarily represent those of their affiliated organizations, or those of the publisher, the editors and the reviewers. Any product that may be evaluated in this article, or claim that may be made by its manufacturer, is not guaranteed or endorsed by the publisher.
